# Sonja Chiappetta, MD, PhD

**DOI:** 10.1007/s11695-020-05175-y

**Published:** 2021-01-03

**Authors:** Rudolf Weiner

**Affiliations:** Sana Klinikum Offenbach, Starkenburgring, 66, 63069 Offenbach am Main, Germany

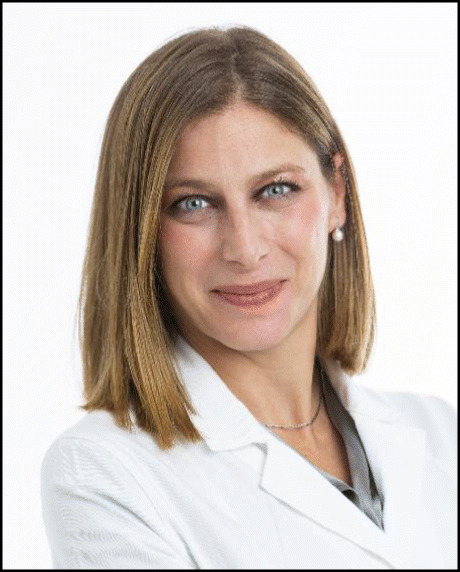



Dr. Sonja Chiappetta is the first female surgeon who leads a center of excellence for bariatric and metabolic surgery in South Italy. Her passion for fighting obesity, her perseverance and persistence during work, her organizing ability and correctness, her enthusiasm for surgery, her scientific curiosity and the opportunity of starting her surgical education with Prof. Rudolf Weiner are all abilities that helped her to become a named young bariatric surgeon in the obesity community.

Dr. Chiappetta was born on 10 February 1984 in Offenbach am Main, Germany. She grew up with love and a persistent support of her parents Elke and Paolo Chiappetta in Heusenstamm, where she graduated high school. She studied medicine in Frankfurt am Main at the Johann Wolfgang Goethe-Universität (2003–2009) and concluded her studies in the top decile of her year. During the entire academic studies, she worked in an emergency medical service and learned the basis of practical medicine.

Due to her love for Italy, Sonja took part in the European program Erasmus and studied 1 year at the University Vita-Salute San Raffaele (2005/2006) in Milan, Italy. As a young student, she was assigned to the surgical department of Prof. Valerio di Carlo (San Raffaele Hospital) and had her first active experience in the operating room. The familiar support of the surgeon colleagues and the high educative surgical standard evolved her passion for surgery.

Back in Frankfurt, she started her first scientific work at the Institute for Diagnostic and Interventional Radiology (Prof. Dr. Thomas J. Vogl), preparing cell lines and operating on rats. She presented the first results of this work, still as a student, at the 89th German Radiology Congress in 2008.

Sonja Chiappetta earned a PhD (magna cum laude) at the University of Frankfurt in 2010. Her experimental thesis was “To evaluate whether tumor volume of hepatic metastasis could be of the predictive value of tumor response to chemotherapy.”

Sonja finished her last part of practical student training in the surgical department of San Raffaele Hospital and met her future husband Dott. Simone Squillante (general surgeon) in the operating room. The young couple decided to start their life in Frankfurt am Main. In 2017, her daughter Paola Chiara Squillante was born. However, Sonja never interrupted surgical and scientific work.

Sonja Chiappetta started her surgical career in 2010 under the leadership of Prof. Rudolf A. Weiner [1], who was at the time the head of the department of general surgery at Krankenhaus Sachsenhausen, Frankfurt am Main, Germany. It was his department gaining both the first national and international Center of Excellence in Bariatric Surgery Certifications in Germany. Until 2006, around 50% of all bariatric procedures in Germany were performed in this hospital. Sonja was one of the youngest surgeons in the long list of bariatric surgeons (W.K. Karcz, M. Daskalakis, S. Weiner, S. Theodoridou, C. Stier, O. Scheffel, M. Frenken and others), which became a leading position.

In 2012/2013, Sonja Chiappetta amplified her surgical training again in Milan, Italy, in the department of general surgery of San Raffaele Hospital under the leadership of Prof. Carlo Staudacher, and highest surgical, medical and scientific education in laparoscopic oncologic surgery, transplant surgery and pancreatic surgery shaped this intense time. Sonja Chiappetta, through knowledge of her bariatric background, noted, presented and published, with the famous pancreatic research group, that duodenal exclusion improves diabetic status even after pancreatic surgery.

In 2014, Sonja Chiappetta returned to Germany, and again joined Prof. Weiner’s team, which back then moved from Frankfurt to Offenbach, just a few miles away, to the largest and most modern hospital in the area at that time. The department for bariatric and metabolic surgery was, with 42 beds, the largest clinic in Germany.

In 2016, Sonja Chiappetta qualified as a specialist in general surgery and gained the position as a staff surgeon. After Christine Stier [2] left the department at the end of 2017, Sonja reached one of the leading positions in the clinic.

With more than 1000 responsible performed surgical procedures, Sonja Chiappetta developed into a specialized and confident surgeon. She became independent very quickly.

Moreover, she developed a special interest in bariatric endoscopy. She performed a large number of interventional procedures like Intragastric Balloon, Overstitch, Endosleeve and others under the guidance of Christine Stier and Prof. Weiner.

Finally, in March 2019, she moved back to Italy and became director of her own bariatric program in Naples. Her bariatric and metabolic surgical unit at Opedale Evangelico Betania gained Center of Excellence Status in February 2020.

Besides clinical work, Sonja Chiappetta was involved in scientific life of our societies and federations at an early stage. Her first presentation at IFSO World Congress was in Hamburg in 2011. She also served as a member of the local organizing committee of congress president Prof. Dr. Rudolf Weiner. Since 2012, she is involved in the organization of the Frankfurt Meeting, as well. In 2015, she was the first female live surgeon during the IFSO World Congress in Vienna. At that time, Prof. Karl Miller was the congress president and Prof. Weiner the IFSO president.

She was a member of the 3rd European Obesity Academy from 2015 until 2017.

Additionally, from 2016 until 2018, she was secretary of the German Society for bariatric surgery (CAADIP), following Sylvia Weiner [5].

Her scientific work was documented in more than 12 registered scientific studies, one highlight of which was the German nationwide register-based cohort study on Edmonton Obesity Score System (EOSS) [3]. In 2019, the scientific cooperation with Christine Stier on thromboprophylaxis yielded them one of the TOP 10 Abstracts of the 24th IFSO World Congress in Madrid. Both were authors of the IFSO statement for indications for surgery of obesity and weight-related diseases [4]. Since 2018, Sonja Chiappetta was the leader of the national postgraduate courses in endoscopic obesity surgery and organizer and live surgeon during the Mini Gastric Bypass course with Robert Rutledge in 2019.

Sonja Chiappetta published more than 50 papers in German and English and wrote 8 book chapters. She is serving as a preferred reviewer for Obesity Surgery, SOARD, Obesity Facts and other journals. She received poster prizes from the German Obesity Society (2016), the German Surgical Society (2018), travel grants from the Germany Society for Visceral Surgery (2017) and the Sana Science Award 2018 in Munich.

Dr. S. Chiappetta began to give lectures and taught general surgery at an early age. She is currently undergoing the academic habilitation process at the Johann Wolfgang Goethe University of Frankfurt.

The line of surgeons who have been trained by me during the last 30 years is long. Many became chief physicians and some of them professors. Sonja is one of the most extraordinary young surgeons I’ve seen. She has an exceptional perception. Her surgical talent is remarkable and she is very hardworking. As a surgeon, she shows great responsibility towards her patients. As a human, she is sincere and honest.

It is therefore a great need for me to describe this unusual development of one of my best surgical fellows. It fills me with pride when I can look back on such successful careers of my surgical fellows, which are now published again in Obesity Surgery. The next generation carries our work into the future.
